# Tips on Establishing a Robotics Program in an Academic Setting

**DOI:** 10.1100/tsw.2006.393

**Published:** 2006-02-17

**Authors:** William D. Steers

**Affiliations:** Department of Urology, University of Virginia School of Medicine, Charlottesville, VA 22908, USA

**Keywords:** robotics, prostate, cancer, technology, economics

## Abstract

Over the past 5 years, robotic-assisted laparoscopic surgery has gone from being a novelty to an accepted approach for intra-abdominal and pelvic surgery. Driving this trend has been the large number of robotic-assisted laparoscopic prostatectomies performed throughout the U.S. Nearly a quarter of the prostatectomies done for prostate cancer in the U.S. in 2006 will use robotic assistance, yet reports fail to confirm cost effectiveness. The most important predictor of a successful program is a champion at the institution. Studies have demonstrated safety and immediate benefits with regard to reduced surgical morbidity such as pain, loss of work, quality of life, and blood loss for a variety of surgeries patients. Specific to prostatectomy for cancer, long-term data on biochemical (PSA) failures and cancer cures, as well as validated secondary outcomes for continence and potency, are still unavailable. Benefits accrue for the surgeon as well with improved ergonomics and potential extension of a surgical career. Yet, enthusiasm for robotics must be tempered by this lack of data and economic limitations. However, if a thoughtful and thorough process in initiating a robotic program is undertaken, the risks to the institution can be minimized. With proper training, the risk to the patient is reduced and with due diligence with regard to market and operative resources, the risk to the surgeon can be eliminated. This report reviews the steps to assess, plan, initiate, and maintain a robotics program at an academic institution with the hope that other programs can benefit from lessons acquired by early adopters of this expensive technology.

## INTRODUCTION

Establishing a robotics program requires more than learning a new surgical technique or how to use a new piece of equipment[1]. Due diligence begins prior to the purchase of a robot. The infrastructure needed to establish a robotics program is substantial and may explain the suboptimal utilization of robotics at some institutions. At the outset, the institution and its surgeons should ascertain whether a program is feasible financially. For both community and academic centers, the clinical and financial outcomes should be monitored. How this will be done must be decided at the outset. This should be followed by a rigorous financial and market analysis. Given the high initial capital costs and recurring direct and indirect outlays, many financially vulnerable hospitals or health systems choose not to start a program. Yet even if not financially viable, hospitals have still pursued robotics in the hope of a multiplier effect and improvement in their operating margin. Likewise, surgeons choose to pursue robotics for a wide array of reasons.

## INITIAL PLANNING

The choice of robot for human intra-abdominal, thoracic, or pelvic surgery is simplified by the fact that there is only one manufacturer. The Zeus device made by Computer Motion is no longer available, leaving the multiarmed DaVinci robot manufactured by Intuitive (Sunnyvale, CA) with a monopoly. Thus, comparative pricing or technological options are unavailable. Unfortunately, such a monopoly may stifle continuous technical innovation and investments in research and development. The Intuitive device represents a significant advance over earlier prototypes with additional capabilities that permitted easier surgery by surgeons without laparoscopic training.

The size of this particular device and skills needed for operation mandate an assessment of operating room (OR) facilities, utilization, and manpower. Because of the current high OR utilization at many sites, the availability of OR facilities and personnel is a barrier to maximizing case load. At academic centers, additional staffing, research capabilities, and considerations for training of residents and fellows need to be considered. Regardless of institutions, a timeline should be established that includes OR modification, equipment acquisition, hiring of personnel, and staff training.

Although this article focuses on urologic applications, a robotic program may be more viable when a multidisciplinary group of surgeons participate and optimize utilization.

### Should You Start a Robotics Program?

A risk/benefit analysis is crucial to the decision whether the patient, the surgeon, or the institution should pursue a robotics program. For patients and surgeons, new medical technology generates varying degrees of enthusiasm and will always attract proponents even before demonstration of improved outcomes. Evidence for improved outcomes based on multiple, prospective, randomized, blinded trials comparing robotic surgery to an open or traditional laparoscopic procedure is nonexistent. Data crucial for patient and surgeon decision making to assess benefits are often limited to retrospective comparisons. Nevertheless, emerging data suggest that the robotic-assisted laparoscopic surgery compared to an open approach reduces analgesia requirements, recovery time, blood loss, and other outcome measures compared to open surgery[2,3,4].

For surgeons, robotic assistance consistently reduces the learning curve for complex laparoscopic procedures, especially involving suturing[4,5]. Another benefit to the surgeon is ergonomics. Open and laparoscopic surgery is associated with physical stressors and difficult posturing leading to degenerative disc disk disease or musculoskeletal problems[6,7]. Blood-borne pathogens are also a concern for OR staff and can be reduced with laparoscopic surgery. Occasionally, a surgeon in mid career may suffer an injury that prevents full range of motion of extremities. Use of a robotic console addresses these concerns and could offer a tangible benefit.

For hospitals, possessing a surgical robot dangles the promise to lure new patients. It can do this in two ways: (1) by attracting new patients specifically for the robot and (2) publicity for the robot and the institution attract a variety of patients who like the idea of receiving care at a technologically advanced facility regardless of whether or not they themselves are being treated with the robot. Hospital administrators nationwide recognize that successful programs all have an identifiable champion. In fact, the lack of a proponent may predict failure of such a complex and costly program.

Teaching hospitals may acquire unique benefits, but also incur substantial challenges. It is the perception by top-quality trainees that the institution with a robotics program is innovative. In 2001, a survey of General Surgery training programs found that up to a quarter of the programs were planning to include robotics as part of their residency and 57% of responders (corresponding to 1,800 surgery residents)

indicated very high interest in robotic surgery, yet only 20% had access to a robot[8]. The number of procedures that can be performed by robotic surgery is growing (Table 1).

**TABLE 1**
**Procedures Performed by Robotic Surgery**

**Cardiac Procedures**
- IMA takedown
- Mitral valve repair
- Epicardial lead placement
- Beating heart TECAB (single vessel)
- Arrested heart TECAB (single and multiple vessel)
- Esophagectomy
- Thymectomy

**General Surgery Procedures**
- Nissen fundoplication
- Gastric bypass
- Heller myotomy
- Colon resection
- Gastroplasty (gastric banding)

**Urology Procedures**
- Prostatectomy
- Pyeloplasty
- Nephrectomy
- Cystectomy

Another institutional benefit is the potential to become a training site or offer telementoring consistent with institutional educational objectives.

## FINANCES

The benefits of robotics must be weighed against significant obstacles and risks. The risk is primarily financial. An initial capital cost of approximately $1.1–1.3 million for the robot itself is a major barrier because recouping this cost is unlikely in the short term. Several studies indicate that owning a Da Vinci robot is not cost effective for the institution. Even if operative times fall to several minutes, one study indicates the program does not pay for itself[9]. Comparison of three surgical approaches for prostatectomy reveals the profit margin is lowest for robotics even if the initial capital investment is ignored[10]. In a preliminary report from William Beaumont Hospital, mean profit from a perineal prostatectomy and open retropubic was $1,560 and $1,050, respectively[10]. Payers do not provide a premium for robotic or sometimes even laparoscopic surgery. Benefactor contributions or leasing are often considered as alternatives to purchasing from a hospital's operating funds. Obtaining a benefactor can shorten the time to obtain a return on investment (ROI) for the institution and helps convince administration. Even then, the recurring costs of robotic accessories, such as disposable robotic attachments, and a yearly maintenance fee of $100,000 requires either increased unit revenue (fee per procedure), incremental business (increased unit volume for higher fixed costs), or usage fees allocated to users. Training costs, both direct and in terms of time away from work, for physicians, nurses, and technicians can be significant. The ability to hire new personnel may be limited. In this era of scarce OR nurses and rationing of OR time, the ability to perform more procedures that may not be profitable is sometimes met with skepticism. Capturing all of these costs and not including the cost of the robot, more than 100 prostatectomies per year from insured patients are needed for the program to break even. The specter of higher deductibles and more patient-incurred financial risks in the future of health care funding could possess significant challenges for costly highly technical therapies such as robotics. Although employers are shifting costs to employees, robotic or laparoscopic prostatectomy may get the employee back to work quicker than open surgery or radiotherapy. However, a comparison with perineal prostatecomy would be enlightening with regard to morbidities and back to work data.

## Market Analysis

Once the program is operational, marketing is a key component with any new procedure or technology. However, the current marketing of robotics has reached peak levels in many markets as evidenced by radio, television, and print advertisements. Reimbursement issues can be substantial if third-party payers refuse to reimburse a premium over open surgery. In this regard, a group from the institution will need to work with third-party payers on contractual arrangements and reimbursement or the data needed for negotiations. Some programs have chosen to put patients at risk for charges and develop alternative programs for the uninsured. This will become increasingly common as employers shift costs to employees. Our department has had the advantage of learning from both successful and failed programs in the U.S. as well as from experience over the past 3 years in a state that, for a population of 7.4 million, has 11 DaVinci robots.

If the viability of the robotic program depends on surgical volumes, the institution must undertake a market analysis of its service area that encompasses disease prevalence and current surgical volumes. Approximately 77,000 prostatectomies for cancer are performed in the U.S. annually. In 2006, it is estimated that roughly 25% will be performed robotically (personal communication, Intuitive Corp). In Virginia, about 5,000 men are diagnosed annually with prostate cancer; about 1,000 undergo radical prostatectomy in the state. If one incorrectly assumes an equal distribution of patients in the state, this translates into at most 100 prostatectomies annually per robot. This represents an amazing shift in approach in the absence of clinical trials demonstrating efficacy comparable to open surgery or radiation therapy. The relationship of surgical volumes to outcomes for surgeries such as radical prostatectomy is well established[11].

Marketing is defined broadly to include advertising in publications or other media, by website, presentations, and notification of referring physicians. For our institution, primary, secondary, and tertiary markets are assessed primarily based on distance from the medical center, but also historical referral patterns. Most institutions will need to recruit patients from beyond their primary service area if increased volumes are needed to cover costs. Reaching beyond historical boundaries raises skepticism and even resentment by regional physicians. On one hand, surgical volumes drive outcomes, so a shift in access could relegate some surgeons to a low volume status. In this regard, being inclusive and offering a training program may be advantageous.

## Operating Room Requirements

The OR planning should include time and room availability, room size, room layout, availability of proper receptacles and circuits, imaging (either monitors or 3-D room projection), and access to supplies. At our institution, at least 580 sq. ft. is required to accommodate the staff, robot, anesthesia chart, table, and 3-D projection system. It is our belief that a dedicated room is optimal to avoid moving the robotic system and risking damage to the mechanical components or wiring. Similar to the experience at Henry Ford Hospital, we have found that a dual digital projection system on a silver-coated screen to allow 3-D viewing by the first assistant facilitates surgery and allows scrub nurses the ability to follow the surgery and anticipate needs. New operating rooms with 3-D projection systems are being designed to aid laparoscopic surgery. Meanwhile, 2-D flat screen video monitors are used by assistants.

Cost effectiveness of robotic surgery is enhanced with reduced operative time and faster turnover[10]. In this regard, any modifications of procedure or technology that lower operative time are essential. Details as small as monitoring % gas remaining in the $CO_2$ tank allow for anticipation of a change, warming of equipment to prevent fogging of the lens, and minimizing retrieval or changing robotic instruments. We have employed an insufflator system with two tanks of gas so that we can switch over to a second tank when the first one gets low and then replace the first empty tank to be ready for use when the second one empties. However, the combination of high insufflation rates and warming may lead to ileus, which we witnessed early in our series if cases are prolonged. We try to maintain enough instruments to perform two cases, which allow back-to-back procedures without having to wait for equipment to be cleaned. For safety reasons, it is optimal to have a backup lens system. However, since we may use a 0° or 30° lens, we chose a backup of only the 0° lens since most or all surgery can be accomplished with this visualization. We also maintain a supply of backup instruments that have been sterilized and are ready to use. Turnover times can be reduced by having a dedicated team that is rewarded for success. In addition, video monitoring of the OR room by a surgeon so that inactivity between cases is observed may help to minimize lost time. Use of a single lens, usually 0°, avoids fogging and time in exchanges. Keeping the lens warm or using an antifog solution (check with Intuitive since not advised for some lenses) is essential to avoiding delays.

## Equipment/Supplies

There are a variety of questions regarding equipment and supplies. What supplies will you require? Where will you get them and how much will they cost? Whose budget will pay for them? How many will you keep in inventory? How many will you have sterile and ready at a given time? Where will they be stored and who will be responsible for managing inventory and reordering? Some items that you want to use may have to be approved by your clinical engineering department. Make yourself aware of existing contracts with vendors that may preclude your ability to purchase from a competitor.

The items needed to support a robotic program, above and beyond what your OR already has available for laparoscopic cases, include hardware obtained from the robot vendor: reusable robotic accessories (e.g., sterile adapters, scopes, light guide cables, trocars); limited life reusable robot arms that can be used on as few as 8 or as many as 20 cases depending on which instrument it is (e.g., needle drivers, forceps, scissors, cautery tools); disposable robotic supplies (e.g., drapes, cannula seals). As the surgical technique has been refined and the surgeon has become more facile, use of only one or two instruments (e.g., curved cautery scissors and bipolar Maryland grasper) has become common, thereby saving disposable costs and time. If one establishes a pediatric robotic program, the need for additional smaller (5 mm) ports and instruments adds additional costs.

In addition to supplies provided by the robot vendor, other supplies include an insufflator. We prefer a heated gas model that is high flow to allow for gas leakage around up to six ports and even the urethra. We also have the ability to switch over to a second tank without losing the pneumoperitoneum. Necessary equipment includes a suction irrigator, a scope warmer, a surgeon’s chair tall enough to see into the surgeon’s console, made of a material suitable for the OR, and video equipment. We use a 3-D projector and portable silver screen. Although a 3-D projection system is not essential, it may help assistants become more facile and keep up with the console surgeon.

## Establishing the Robotic Team

Robotic surgery is labor intensive in terms of OR personnel. At least two surgeons, a scrub nurse, and an anesthesiologist are required. Usually, a second assistant is necessary, but can be replaced in some instances by a fourth arm option available on the Da Vinci robot. The need for a very highly skilled assistant may be a significant barrier to a small private practice group entering the robotic arena. The bedside surgeon must be a fully trained laparoscopic surgeon or surgical technician. Unlike open or other endoscopic surgeries, many robotic surgeries, especially radical prostatectomy, are nearly impossible without an accomplished first assistant[15,16]. The team needs to decide whether the first assistant is a resident, fellow, faculty member, or surgical technician. This manpower and training decision is complex and has implications for departmental morale as well as financial implications. Moreover, one scrub nurse may be insufficient to handle imaging, trips to the supply room, or communication with the company if a malfunction occurs. Given the current plethora of robots, a robotic company representative is now rarely present, often needed as sites began their programs. If many new patients are attracted to the institution, additional secretarial and office staff may be necessary. Our adult and pediatric robotic team, excluding residents, amazingly includes 16 people.

A dedicated team of surgeons and nurses is crucial to the successful implementation of robotics. Constantly changing assistants, nurses, and anesthesiologists delays start times, turnover, and operative time.

Defining goals, obtaining sufficient training, holding team events such as dinners, and encouraging joint continuing medical education programs will ease the stress when cases go overtime or problems arise. Continual communication among the team with newsletters and follow-up of patients is helpful to maintain esprit de corps. The start-up costs above the capital equipment acquisition and inventory expenses associated with the establishment of a robotics program are summarized in Table 2. This amount of 3-year funding is useful to ensure that a dedicated team is built and maintained, in addition to funding for the critical training and marketing expenses essential for program success.

**TABLE 2**
**Robotics Program 3-Year Projected Additional Expenses (Exclusive of Robot, Supplies, and Instruments)**

| | |
|---|---|
| 3-D imaging equipment | $35,000 |
| Additional robotic operating expenses: | |
| Personnel | |
| RN salary and benefits | $230,000 |
| Prog. Tech/Proj. Coord. salary and benefits | $230,000 |
| Administrative support salary and benefits | $60,000 |
| Statistician support | $40,000 |
| OTPS expenses | |
| Training | $100,000 |
| Marketing | $200,000 |
| OR team CME and meetings | $60,000 |
| Service agreements | $250,000 |
| Total | $1,205,000 |

It may be beneficial to have urology subspecialists focusing on a specific surgery. Problems with positioning, technical nuances, and training time may be minimized by specialty teams. At our institution, we find robotic surgery facilitates any laparoscopic surgery that involves suturing due to the proprietary wrist range of motion of the Da Vinci system. Ureteropelvic junction reconstruction, urinary diversion/augmentation, and reimplantation are performed[17]. The advantages for pelvic surgeries such as radical prostatectomy and cystectomy, as discussed below, are noteworthy[3,15]. Less clear are other pelvic surgeries such as pelvic lymphadenectomy, sacrocolpopexy, and hysterectomy. We have found that the laparoscopic approach is easier than traditional methods in patients who have undergone bilateral inguinal mesh herniorrhaphies because less dissection is necessary and exposure is improved without retraction. Blood loss is dramatically less because pneumoperitoneum reduces venous oozing. Less venous bleeding requires less cautery and subsequent tissue injury. Postoperative pain occurs less than with open surgery, not only because of less retraction and shorter incisions, but because inflammatory cytokines (interleukins) and nociceptive compounds (bradykinins) are reduced with $CO_2$ pneumoperitoneum[18,19]. Probably the greatest advantage is the dramatic improvement in visualization in 3-D, not achieved with traditional laparoscopic cameras. This visualization may allow better preservation of neurovascular and muscular structures. In our opinion, what is lost by a lack of tactile feedback is more than compensated for by improved visualization.

The adoption of robotic surgery by other surgeons helps to ensure success of the program, although it limits urologic use. Originally developed for cardiovascular surgery, robotics has been used by general surgeons, microsurgeons, head and neck surgeons, and gynecologists. Because the general impression is that an identified champion is required for success of a robotic program, having other vested surgeons helps to ensure longevity and innovation.

## Training

One of the highest barriers to laparoscopic surgery has been the amount of training necessary to acquire skills and perform increasingly complex surgery. Robotics reduces the learning curve for laparoscopic surgery[20]. Collective experience shows that the surgeon at the console need not have trained as a laparoscopic surgeon if there is a capable assistant at the bedside[5]. In fact, unlike skilled laparoscopic surgeons, the laparoscopically naïve surgeon at the console need not unlearn certain skills such as suture tying in 2-D. Nonetheless, extensive training is needed beyond a short (several days) course and should not be underestimated.

Short courses including an animal lab and use of models may familiarize the console and bedside surgeons and team with the equipment, general principles, and techniques, but repetition of actual surgery is undeniably the best approach. On-site mentoring is often not feasible and this would seem to necessitate a sabbatical at a high-volume center. Proctoring for the first several surgeries is highly advisable. The indirect costs of training must be factored into the business plan and the career aspirations of the surgeon need to be taken into account.

Surgical training consists of two separate, but equal components: use of the robot and the surgical procedure. Training on the robot using models is required to learn the response of the robotic arms and suturing. Laparoscopic training includes specialized port placement and other techniques used by the bedside team. Proper patient positioning and port placement are crucial. Training in surgical technique begins with porcine surgery, review of videos, watching numerous procedures, and then completing various steps of the procedure before attempting an entire case. The transition from animal surgery to humans is problematic because of subtle differences in anatomy, including tissue planes and amount of adipose tissue, not to mention the controversy surrounding animal experimentation. Just as important as training on the console is the training of the laparoscopic cosurgeon. This individual needs to know and anticipate each step to facilitate surgery and reduce operative time. Slow adaptation to robotics and lengthy operative times may result from a lack of skilled cosurgeons.

Optimal rotation time for trainees previously exposed to laparoscopic surgery on robotics should be determined, but our bias is that at least 6 months is necessary. A training program for residents is structured to include robot technical detail proficiency, robot technique practice, animal lab, emergency exercises, and laparoscopic simulation. In the absence of objective data, we prefer a trainee to second assist in at least 10 cases before first assisting, then first assisting in 20 cases before time at the console. The trainee proceeds with only a limited portion of the procedure and eventually should practice the individual steps (e.g., retroperitoneal exposure, apical suture, anastomosis) before being allowed to proceed with the entire procedure. Conversely, the inability of the attending teaching surgeon to proceed with surgery in the case of a floundering trainee is extremely limited, thus making robotic training the most difficult of all surgical procedures in our armentarium. Moreover, patients often demand that no trainees participate in their procedure, yet it must be explained that this is team procedure. It remains to be seen whether robotic surgery especially radical prostatectomy as practiced today is best a resident or a fellow procedure. This decision depends on the individual talents of the resident, surgical volumes, and ability to staff the bedside, as well as surgical time available.

There is growing recognition that surgeons in training will be required to utilize simulated surgery prior to operating on patients. Clinical competencies for residency training require objective and quantifiable measures of procedural skills. This alone will force the adoption of surgical simulators similar to those used to train pilots and other professionals. Endoscopic simulators such as the UroMentor (Symbionix) have advanced sufficiently to become important training tools in residency training programs[21]. Robotic surgery lends itself to simulated learning better than open surgery does. The development of a virtual reality training system for robotics would lessen the learning curve and help the rapid adoption of this technology by allowing quantification of skills[22,23].

The full benefits to establishing a robotics program have yet to be realized. One unforeseen and unproven advantage of robotics may be obtaining more uniformity in surgical outcomes. Whereas manual dexterity and other skills can be acquired, differences between surgeons still exist in some cases. A platform that gives the same vision, scaling, antitremor, and device capabilities to all surgeons has the potential to raise the overall level of expertise. To use the analogy of fighter pilots, in World War I the aces had the quickest reactions and best eyesight to see combatants in the distance. Today, advanced capabilities of fighter jets have raised the level of performance of the entire cadre of pilots.

The cost and time involved in training a surgeon are enormous. Often surgeons reach peak performance in their fifth decade of life. It is rare, but not unheard of, for a surgeon to sustain an injury, such as a cervical disk herniation, that limits or ends a surgeon's operative career. This surgeon could still potentially perform surgery using robotic technology.

The economics of robotics could also be justified if it allows a group of surgeons at an institution to avoid years of retraining to acquire advanced laparoscopic skills necessary to perform complex laparoscopic surgery. If, for example, it takes at least 2 years and several hundred surgeries to become proficient in laparoscopic prostatectomy, the lost revenue and time to a mature surgeon to take an extensive sabbatical to learn the technique may start approaching the price of the robot.

In the future, telesurgery where the surgeon at a regional hospital is assisted by the robotic center may be feasible, especially in remote areas[12,13]. Our institution serves a predominately rural population with limited access to tertiary health care and patients must often travel 2 to 6 h to reach our center. Telerobotic surgery, with training of rural surgeons to assist locally, is an attractive solution.

## ESTABLISHING A TIMELINE

The timeline to achieve various landmarks will vary among institutions. It is reasonable to plan for 6–12 months to enable financing, 1–3 months for training, 1–3 months for OR renovation, and 2–4 weeks for installation. Expect 6–12 months to ramp up the program, which may plateau 12–18 months later. Some programs at that time may then contemplate expansion with another robot if enough surgery is performed, or for development of a training program. Realistically, the development of a robotics program is measured in years, not months.

## RESEARCH ISSUES

Some have argued that robotics is a technology looking for a use. Using a robot for some procedures is similar to driving a Lamborghini in second gear. The full realization of its potential is still unknown. Academic medical centers should actively engage in research in cooperation with industry and put themselves in a position to take robotics to the next level. Preliminary reports on using the Da Vinci robot to perform tedious sural or genitofemoral nerve grafting after prostatectomy recently appeared[24,25]. It may become feasible to perform bladder transplantation with nerve grafting. Preprogramming specific operative steps based on fixation of tissues and intraoperative imaging with the establishment of 3-D coordinates may be an attainable goal.

Beyond outcome research, we are pursuing advanced bioimaging, telerobotic mentoring and surgery, and expansion of surgical indications. In animal studies, we have been able to visualize prostate cancer and cavernous nerves real time, *in vivo* to a resolution of 40 μm using a laser confocal microscope. Cancer can be visualized using antibodies to 100 kD type 2 membrane protein, prostate-specific membrane antigen (PSMA), while retrograde axonal tracers identify nerves supplying the penis (Fig. 1). Furthermore, we can image neuronal function allowing assessment of real-time injury to nerves. For the first time, the robotic platform combined with bioimaging provides the exciting opportunity to perform cellular surgery and transform surgical practice. Once this has been shown to be safe and effective in humans, robotic surgery will become essential to minimize positive tumor margins or injury to microscopic structures instead of a novelty or expensive marketing ploy. We can expect novel uses to appear over time, given sufficient interest and research funding.

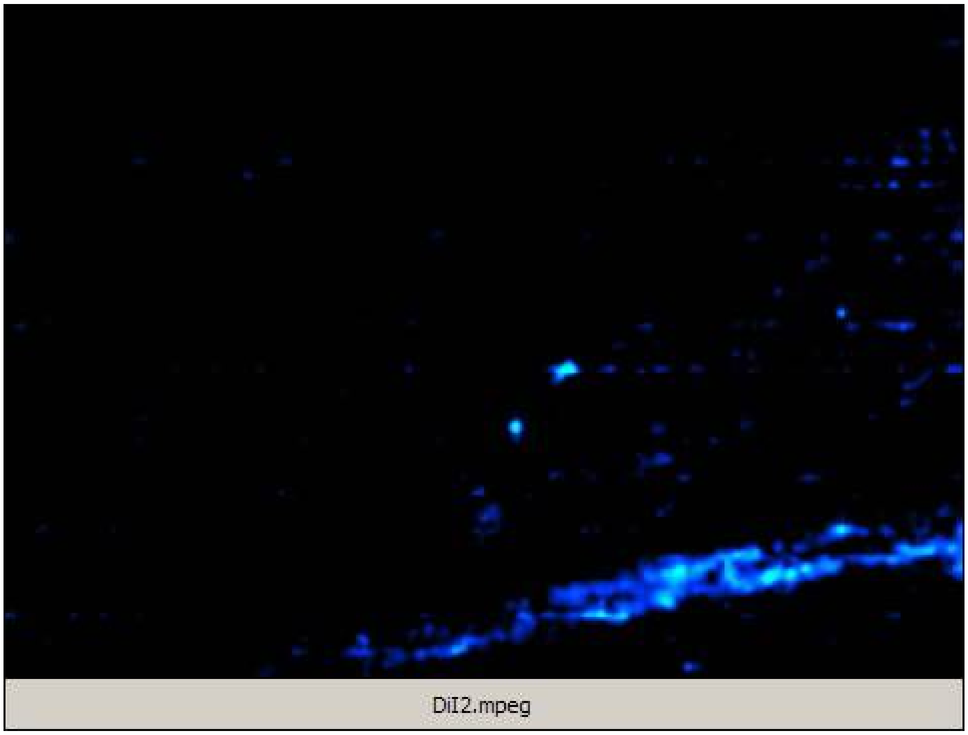

**FIGURE 1.** Captured video image of rat cavernous nerve using confocal laser microscope 1 week after injection of penis with retrograde axonal tracer.

## MONITORING OUTCOMES AND SUCCESS

The implementation of a robotics program should include defining specific measurable objectives for caseloads to be attained over the baseline volumes. The metrics to evaluate the success of the program would be surgical volume compared with baseline. Outcome measures specific to the procedure such as continence, potency, blood loss, analgesic requirements, tumor margins, and prostate specific antigen (PSA) levels following prostatectomy at various intervals must be assessed. Additional quality assessment is based on (1) customer satisfaction, (2) performance over time against University Health Care Consortium (UHC) and national benchmarks, (3) performance over time on internally designed quality performance measures, and (4) overall quality of life instruments.

Customer satisfaction includes both referring MD satisfaction and patient satisfaction. Both should be surveyed qualitatively and robotic surgery patients should be surveyed specifically for their satisfaction with their surgery. Our initial patient satisfaction assessment has been qualitative in nature and shows a majority of satisfied patients. Feedback from these patients is being used to develop a patient satisfaction survey. We ask patients, following their surgery, if they were satisfied about our ability to provide accurate and complete information on what to expect, convenience of scheduling, and minimizing the number of trips to the Medical Center throughout the process. We ask them how user friendly our system is for calling and asking clinical, logistical, or financial questions. Performance over time against benchmarked data for morbidity, including complications, average length of stay, charges, cost per case, and tumor margins should all be evaluated. We have captured some of these items. For example, for our initial 12 robotic radical prostatectomies, hospitalization averaged 1.5 days, average blood loss was 215 cc, and pain levels were between 0 and 2 (on a scale from 1–10 with 10 being the most pain) on the day following surgery and 0 on the date of discharge.

Performance over time on internally designed quality performance measures will test whether the combination of robotic surgery with emergent biomedical imaging modalities will compensate for the lack of haptic feedback. In future studies, outcomes will be measured and analyzed to assess this hypothesis. Ultimately, the success of a robotics program depends on adequate surgical volumes with frequent cases to maintain skills sets of the robotic team. For our program, this translates into cases every week.

## BIOSKETCH

**Dr. Steers** received his medical degree in 1980 from the Medical College of Ohio after receiving a degree in Chemical Engineering from Cornell University. Following a residency at the University of Texas and M.D. Anderson Hospital, Dr. Steers completed a fellowship in Neurobiology and Pharmacology at the University of Pittsburgh. He has been at the University of Virginia since 1988 and became Chair of the Department of Urology in 1995. Dr. Steers is a trustee of the American Board of Urology and is Principle Investigator on two NIH grants. Research interests include neurophysiology, neuropharmacology, incontinence, and erectile dysfunction in addition to robotic surgery.
